# Parent-child communication and inner dialogues in the self-awareness of children with disabilities

**DOI:** 10.1192/j.eurpsy.2021.1355

**Published:** 2021-08-13

**Authors:** N. Burlakova, O. Karpova

**Affiliations:** Department Of Neuro- And Pathopsychology, Lomonosov Moscow State University, Moscow, Russian Federation

**Keywords:** parent-child communication, children with disabilities, self-awareness, inner dialogues

## Abstract

**Introduction:**

Analysis of problems arising in communication between parents and their children with disabilities is a part of biopsychological examination of disease.

**Objectives:**

The study was aimed at exploring the parent-child communication in children suffering from chronic neurological disorders in order to organize the optimal psychological rehabilitation.

**Methods:**

The study consisted of two stages: 1) CAT (Bellak) and drawing tests, performed by child; 2) experiment involving both parent and child, making up a story together (CAT-H, parallel to the task performed by the child). Parents filled in a questionnaire on their communication with the child; its results were compared to the situations of real communication. Other methods used included: observation, analysis of family situation and child’s development; coding of communicative elements; analysis of the story by the method by N. Burlakova (Burlakova, 2001). The study involved 34 persons: 17 children (aged 7–10) + 17 parents (15 mothers, 2 fathers). The dyads were studied when the children received treatment in the hospital (resided in the hospital together with the parent).

**Results:**

1) Activity of the child together with the parent revealed several communicative patterns, which correlated differently to the estimation of communication by the parent. 2) The types of inner dialogues in children were discovered, which gave concrete expression to the inferiority feeling caused among others by the communication in the parent-child dyad. 3) The organization of the research enabled to follow the “production” of dialogues of self-awareness in children with chronical diseases.

**Conclusions:**

The conducted research enables organization of individualized psychological and psychotherapeutical aid.

